# A 12‐genus bacterial signature identifies a group of severe autistic children with differential sensory behavior and brain structures

**DOI:** 10.1002/ctm2.314

**Published:** 2021-02-19

**Authors:** Kai Shi, Lingli Zhang, Juehua Yu, Zilin Chen, Senying Lai, Xingzhong Zhao, Wei‐Guang Li, Qiang Luo, Wei Lin, Jianfeng Feng, Peer Bork, Xing‐Ming Zhao, Fei Li

**Affiliations:** ^1^ Institute of Science and Technology for Brain‐inspired Intelligence Fudan University Shanghai China; ^2^ School of Mathematical Sciences, SCMS, and SCAM Fudan University Shanghai China; ^3^ College of Information Science and Engineering Guilin University of Technology Guilin China; ^4^ Department of Developmental and Behavioural Pediatric & Child Primary Care Xinhua Hospital Shanghai Jiao Tong University School of Medicine Shanghai China; ^5^ Brain and Behavioural Research Unit of Shanghai Institute for Pediatric Research and MOE Shanghai Key Laboratory for Children's Environmental Health Xinhua Hospital Shanghai Jiao Tong University School of Medicine Shanghai China; ^6^ NHC Key Laboratory of Drug Addiction Medicine (Kunming Medical University) First Affiliated Hospital of Kunming Medical University Kunming Yunnan China; ^7^ Collaborative Innovation Center for Brain Science Department of Anatomy and Physiology Shanghai Jiao Tong University School of Medicine Shanghai China; ^8^ Research Institute of Intelligent Complex Systems Fudan University Shanghai China; ^9^ European Molecular Biology Laboratory Meyerhofstraße 1 Heidelberg Germany; ^10^ MOE Key Laboratory of Computational Neuroscience and Brain‐inspired Intelligence, and Frontiers Center for Brain Science Shanghai China

Dear Editor,

Autism spectrum disorder (ASD) refers to a group of heterogeneous neurodevelopmental disorders with neuropsychological and behavioral deficits,^1^ whose accurate subtyping is important but no efficient method is available.^2^, ^3^ Emerging studies suggest a possible mechanism that involves the microbiota–gut–brain axis^4^, ^5^; however, the associations underlying the gut microbiota community, brain structure,^6^ and behavioral symptoms are poorly defined in ASD.

Herein, we aimed to investigate and identify a potential microbial signature that is linked to brain structure variations and severity of symptoms in patients with ASD by using a machine learning framework.^7^ All methods are fully detailed in Materials and Methods in the Supporting Information. Among 128 eligible patients recruited, the initially collected cohort was used as the discovery set (*n *= 78), and the subsequently collected cohort was used as the test set (*n* = 50). The clinical characteristics including age, gender, and CARS^8^/ADOS^9^ total scores were well balanced (Table 1). Two other public sets were used as the validation sets (SRP093968, PRJEB15418).

**TABLE 1:** Clinical characteristics of subjects in the discovery and test sets, and behavioral differences between the two ASD subpopulations

		Discovery set	Test set	
		Xinhua ASD registry (*n* = 78)	Xinhua ASD registry (*n* = 50)	*p* ^a^ value
Characteristics	Age (year)	4.8 (3–12)	5.2 (3–12)	0.25
	Gender			
	*Female*	12 (15.4%)	5 (10%)	0.38
	*Male*	66 (84.46%)	45 (90%)	
	ADOS			
	*Total score*	16.1 ± 3.3	16 ± 4.3	0.94
	CARS			
	*Total score*	37.3 ± 3.6	36.9 ± 4.8	0.42
	*Item scored ≥3*	4.99 ± 3.21	4.51 ± 3.40	0.76
	Disease severity			
	*Severe*	36 (46%)	16 (32%)	0.11
	*Mild to moderate*	42 (54%)	34 (68%)	

		Discovery set				Test set			
		**ASD^mp1^**	**ASD^mp2^**	$\varepsilon^{2}$	***p*‐value**	**ASD^mp1^**	**ASD^mp2^**	$\varepsilon^{2}$	***p*‐value**
Behavior symptom	Disease severity								
	*ADOS total score*	15.4 ± 3.4	17.3 ± 2.9	0.08	0.012*	15 ± 4.5	17.7 ± 3.2	0.08	0.043*
	*CARS total score*	36.2 ± 3.7	39 ± 3.4	0.15	0.001*	35.8 ± 4.4	39 ± 4.8	0.09	0.032*
	*Item scored ≥3*	4.1 ± 2.8	6.4 ± 3.4	0.10	0.0058*	4.1 ± 3.4	6.9 ± 4.1	0.12	0.012*
	Functional impairment								
	*Social impairment*	24.3 ± 2.4	26.1 ± 2.2	0.14	0.0026*	23.9 ± 3.2	26.7 ± 3.1	0.12	0.03*
	*Distorted sensory response*	7.1 ± 0.97	7.6 ± 1.1	0.06	0.048*	6.8 ± 1.4	8.1 ± 1.2	0.16	0.009*
	*Negative emotionality*	7.0 ± 1.1	7.5 ± 1.0	0.07	0.06	7.1 ± 1.1	7.4 ± 1.5	0.01	0.8

CARS total score is the sum of 15 subscale scores. cars1: human relationships; cars2: imitation; cars3: affect; cars4: use of body; cars5: relation of objects; cars6: adaptation to change; cars7: visual responsiveness; cars8: auditory responsiveness; cars9: near receptor responsiveness; cars10: anxiety reaction; cars11: verbal communication; cars12: nonverbal communication; cars13: activity level; cars14: intellectual consistency; cars15: global impression. Social impairment is the sum of 11 subscale scores, including cars1, cars2, cars4, cars5 cars10, cars11, cars12, cars13, cars14 and cars15. Negative emotionality is the sum of 3 subscale scores, including cars3, cars6, and cars10. Distorted s*ensory response* is the sum of 3 subscale scores, including cars7, cars8, and cars9. The quantitative measurements are presented as mean ± standard deviations. The group differences are evaluated with the Kruskal–Wallis test for continuous variables, and the *p*‐value is based on 5000 random permutations. CARS, Children Autism Rating Scale; ADOS, Autism Diagnostic Observation Schedule.

First, we developed a computational pipeline utilizing a greedy search strategy and identified a microbial signature of 12 bacterial genera in the discovery set, by which the subjects were classified into two subpopulations, denoted as ASD^mp1^ and ASD^mp2^ (Figure 1A and B). We subsequently trained a k‐nearest neighbors (kNN) classifier using the abundance profile of the 12 bacterial genera, which performed very well with the classification accuracy of 0.87 in leave‐one‐out cross‐validation (Figure S1).

**FIGURE 1:**
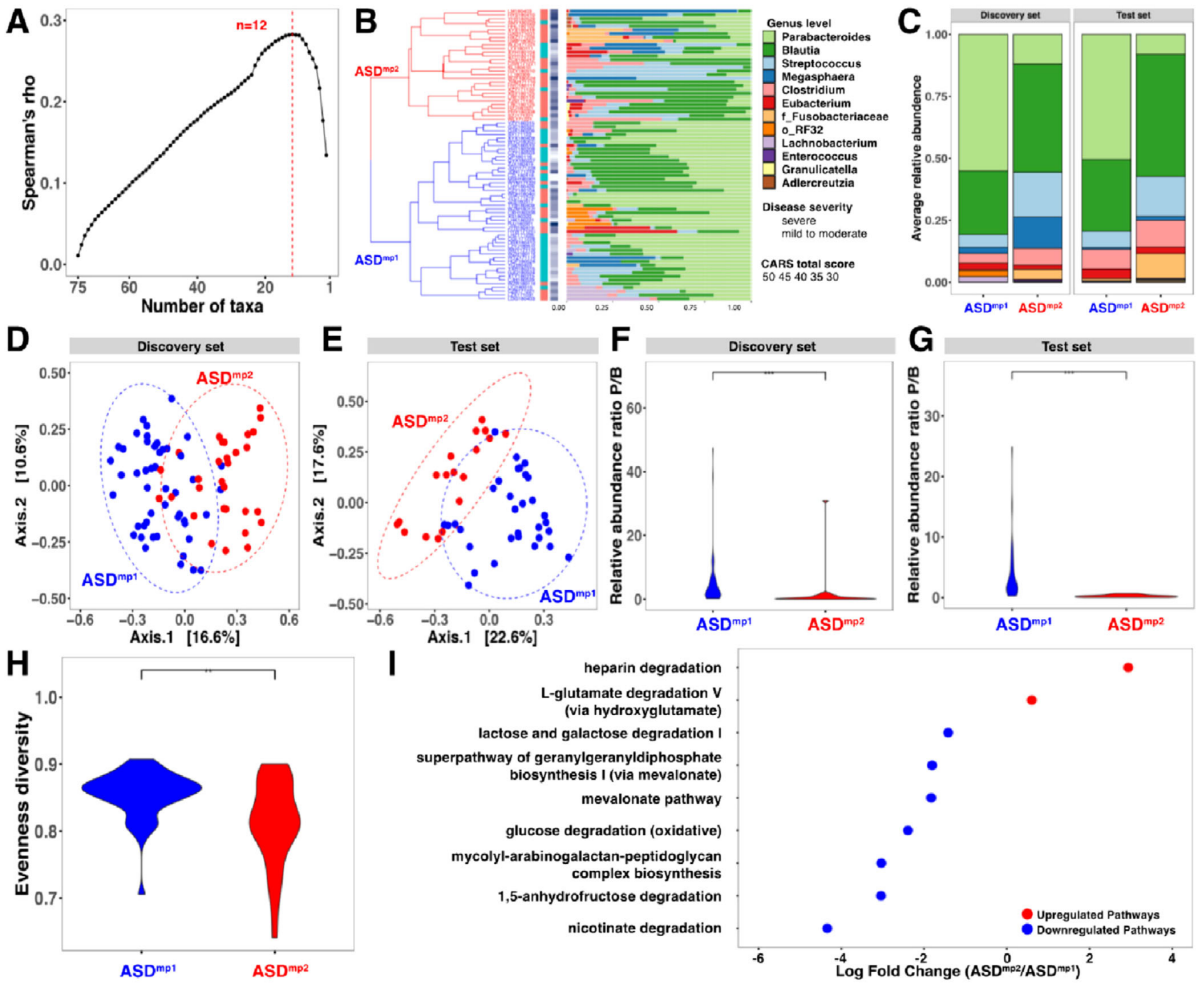
Distinct microbial patterns in subpopulations of children with ASD. (A) A signature of 12 genera that was most associated with the behavioral characteristics using Spearman correlation. (B) Two ASD subpopulations with a 12‐genus signature in the discovery set. Samples were categorized by hierarchical clustering using average linkage with the similarity of 12 genera on the Bray‐Curtis metric. The first color bar indicates the severity of the disorder (CARS total score: red ≥ 36, blue < 36), and the second color bar shows the CARS scores. (C) Stacked horizontal bar charts depict the variability in genus‐level composition for individuals by groups for 12 microbial markers. (D and E) Principal component analysis (PCoA) of a 12‐genus signature based on Bray‐Curtis dissimilarity for the discovery and test sets. (F and G) The comparison of the relative abundance ratio between *Parabacteriodes* and *Blautia* in the discovery and test sets. (H) α‐Diversity in ASD^mp1^ and ASD^mp2^ as measured by observed amplicon sequence variants. (I) Differential MetaCyc pathway analysis as calculated by edgeR. Fold change (ASD^mp1^/ASD^mp2^) as a factor for Benjamini–Hochberg corrected *p*‐values. CARS, Children Autism Rating Scale; ASD^mp1^, ASD subpopulation 1; ASD^mp2^, ASD subpopulation 2

The diversity analyses of the gut microbiome indicated that the two subpopulations ASD^mp1^ and ASD^mp2^ were significantly distinguishable (Figure 1C–E). The alpha diversity of the gut microbiome in the subjects of ASD^mp1^ was much greater than that of ASD^mp2^ (*p*  =  0.0017, Figure 1H) with respect to Pielou's evenness diversity index. Similar results were observed for the other diversity measures: Shannon's entropy (*p *= 0.016), Faith's phylogenetic diversity (*p *= 0.04), and the number of observed OTUs in the discovery set (*p *= 0.05, Figure S2). Among the 12 genera, three displayed a significant differential relative abundance between two subpopulations in both discovery and test sets (Table S1): *Parabacteroides* (*p *=  $8.03\;\times 10^{-9}$)*, Streptococcus* (*p *= 0.03), and *Granulicatella* (*p *= 0.021). Interestingly, the opposite abundance pattern of *Parabacteriodes* and *Blautia* was observed ($\chi^{2}\;$= 29.6, *p *= $5.12\;\times 10^{-8}$, Figure 1F). We then performed MetaCyc Pathway analysis and identified a list of nine differential metabolic pathways (fdr.*p *< 0.05), in which seven were enriched in ASD^mp1^, such as nicotinate degradation (fdr.*p *= $3.44\;\times 10^{-9}$), glucose degradation (fdr.*p *= 0.012), and mevalonate (fdr.*p *= 0.012) pathways. On the contrary, heparin (fdr.*p *= 0.00039) and l‐glutamate degradation (fdr.*p *= 0.04) were enriched in ASD^mp2^ (Figure 1I). (the FDR adjusted *p* value: *fdr.p*; the permuttion adjusted *p* valure: *perm.p*)

In parallel, the two subpopulations that were identified by the 12‐genus signature also displayed significant differences in the CARS total score (perm. *p *= 0.001), ADOS total score (perm. *p *= 0.012), and CARS item scores ≥3 (perm. *p *= 0.0058) in the discovery set (Table 1). The proportion of subjects with severe symptoms in the ASD^mp2^ was significantly higher than that of the ASD^mp1^ (42% versus 77%, odds‐ratio = 0.2, *p *= 0.005).

Using the *k*NN classifier previously trained, the subjects in the test set were also classified into two subpopulations, ASD^mp1^ (*n* = 32) and ASD^mp2^ (*n* = 18). Similarly, the differences between the two subpopulations in the test set were observed in CARS total score (perm. *p *= 0.032), ADOS total score (perm.*p *= 0.043), CARS item score ≥3 (perm.*p *= 0.012), and microbial profiles (Figure 1C–E). An opposite abundance of *Parabacteriodes* and *Blautia* was also observed ($\chi^{2}$ = 27.3, *p *= $1.7\;\times 10^{-7}$, Figure 1G). The proportion of severe subjects in the ASD^mp2^ was much higher than that in the ASD^mp1^ (41% versus 78%, odds ratio = 0.2, *p *= 0.018).

Furthermore, the *k*NN classifier was applied to the two geographically separate sets. Although the microbiota diversity from individuals with a geographical difference was large, the 12‐genus signature was able to categorize the subjects into the ASD^mp1^ and ASD^mp2^. The *Parabacteriodes* genus displayed a higher average abundance level in ASD^mp1^ as compared with that of ASD^mp2^ (Figure S3), which was consistent with the previous results.

We further explored the behavioral measurements in the three primary CARS domains^8^ representing diverse behavioral and emotional aspects between these two subpopulations. Compared to ASD^mp1^, the subjects in ASD^mp2^ displayed markedly higher scores on the CARS domains of *social impairment* (perm. *p *= 0.0026) and *distorted sensory response* (perm. *p *= 0.048), and also indicated a trend in *negative emotionality* in the discovery set (perm. *p *= 0.06) (Table 1). Consistently, the comparison in the test set revealed significant differences in *social impairment* (perm. *p *= 0.03) and *distorted sensory response* (perm. *p* = 0.009), suggesting that the unique microbial profile is correlated with severity of the disorder at specific behavioral and emotional domains in human.

Meanwhile, we sought to determine whether dysbiosis, gastrointestinal dysfunction, and feeding behavior assessments are differentiated between the two subpopulations. By combining the discovery and test sets, we explored dietary and gastrointestinal assessments in 75 subjects (ASD^mp1^: *n* = 47 versus ASD^mp2^: *n* = 28). Although we observed more constipation in severe ASD patients (ASD^mp1^:29/47, ASD^mp2^:20/28, *p *= 0.45), there was no significant difference in gastrointestinal symptoms between the two subpopulations. Notably, significant differences in feeding behavior “narrowed food spectrum” (*p *= 0.044) and “resistance to accept new food” (*p *= 0.031) were identified between the two subpopulations, suggesting that there were indeed more eating problems in patients with severe ASD and perhaps the severity of autistic feeding behavior may lead to altered microbial composition in ASD patients.

To better understand how the signature of 12 genera affects the autistic behavior through the microbiota–gut–brain axis, we compared the brain structures between the subjects from ASD^mp1^ (*n* = 62) and ASD^mp2^ (*n* = 37) by combining the discovery and test sets. Brain volumetric differences between the subjects from the two subpopulations were observed in 12 regions (Figure 2A, Table S2), which spread across primary sensory regions, such as *superior occipital gyrus*, *middle occipital gyrus*, *superior temporal gyrus*, and *postcentral gyrus*. Significant volumetric differences were also observed in PCC, *cuneus cortex*, *calcarine gyrus*, *supramarginal gyrus*, and superior *parietal gyrus*. Interestingly, the PCC volume was negatively correlated with the autistic symptoms related to the *distorted sensory response* (*r* = −0.268, perm. *p* = 0.033, Figure 2B) in the ASD^mp1^. Similarly, a negative correlation was observed in the left *superior temporal gyrus* (*r* = −0.287, perm. *p* = 0.030) and right *middle occipital gyrus* (*r* = −0.260, perm. *p* = 0.049) with autistic sensory scores (Figure 2B).

**FIGURE 2:**
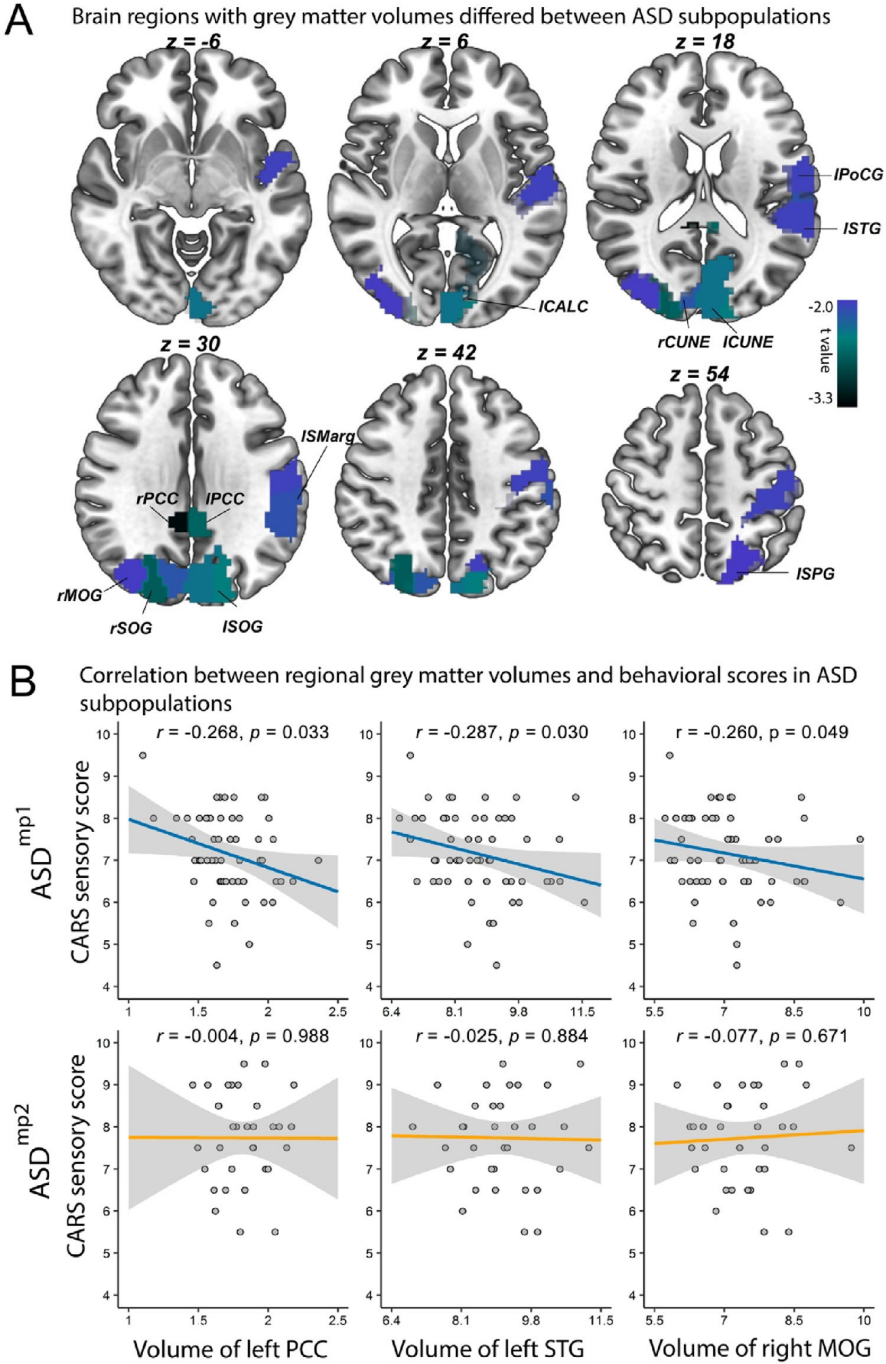
The differential brain image regions between ASD patients from subpopulations. (A) Volume differences between subjects in ASD^mp1^ and ASD^mp2^, defined by the 12‐genus signature. (B) Association between left PCC, left STG and right MOG volume and CARS sensory score of subjects in ASD^mp1^ and ASD^mp2^. Abbreviations: lPCC, ‐ left posterior cingulate cortex; rPCC ‐ right PCC; CUNE‐cuneus cortex; SOG, superior occipital gyrus; MOG, middle occipital gyrus; PoCG, postcentral gyrus; STG, superior temporal gyrus; SPG, superior parietal gyrus; SMarg, supramarginal gyrus; CALC, calcarine gyrus; CARS, Childhood Autism Rating Scale

Taken together, to our best knowledge this is the first report demonstrating a potential microbial signature associated with brain structure variations and behavioral characteristics in ASD, which would further strengthen our understanding of this severe and heterogeneous neurodevelopmental disorder. However, more research with a large cohort study to clarify the role of the influence of dysbiosis on brain function and autistic behavior is needed in the future.

## CONFLICT OF INTEREST

The authors declare that they have no conflict of interest.

## ETHICS APPROVAL AND CONSENT TO PARTICIPATE

This study was approved by the Institutional Review Board of the Xinhua Hospital Shanghai Jiao Tong University (XHEC‐C‐2019‐076). The study was performed in accordance with the Helsinki Declaration and Rules of Good Clinical Practice (GCP). Written informed consents were signed by the caregivers of all applicants after the study protocol was fully explained.

## AUTHOR CONTRIBUTIONS

Kai Shi, Lingli Zhang, and Juehua Yu designed the study, analyzed the data, and wrote the manuscript. Zilin Chen, Shenying Lai, Wei‐Guang Li, and Qiang Luo analyzed the data and helped design experiments. Fei Li and Xing‐Ming Zhao designed the study, supervised all work, and helped write the manuscript.

## DATA AVAILABILITY STATEMENT

Data are available on a reasonable request from the corresponding author.

## Supporting information



Supporting information
