# Supplementary material for: A 12‐genus bacterial signature identifies a group of severe autistic children with differential sensory behavior and brain structures
Source: Clin Transl Med. 2021 Feb 19;11(2):e314. doi: 10.1002/ctm2.314 (PMC7893807; doi:10.1002/ctm2.314)
Supplement: Supplementary file 1 — Supporting information [file CTM2-11-e314-s001.docx]

**MATERIALS AND METHODS**

**Ethics statement and patients**

The ASD patients, aged 3-12 years old, were recruited from the Xinhua ASD registry at Xinhua Hospital in China. Comprehensive behavioral assessments, dietary and gastrointestinal symptoms assessment, collections of feces samples, and T1-weighted brain structural imaging were performed for all patients. The patients were diagnosed with ASD according to the Diagnostic and Statistical Manual of Mental Disorders, Fifth Edition (DSM-5). Diagnoses were confirmed with the Autism Diagnostic Observation Schedule (ADOS), and a Children Autism Rating Scale (CARS) total score of no less than 30.

Patients were excluded if they were currently using antibiotics or probiotics or had taken them within the last 2 weeks, or had special dietary restrictions (e.g. casein, gluten), or had any reported structural abnormalities in the brain. Of the 128 patients that were recruited, the initially collected cohort was used as the discovery set (n = 78), and the subsequently collected cohort was used as the test set (n = 50). All procedures for our study were approved by the Research Ethics Committee of Xinhua Hospital affiliated with Shanghai Jiao Tong University School of Medicine (XHEC-C-2019-076). Written informed consent was obtained from the parent or legal guardian of each participant.

**Behavior** **scale, dietary and gastrointestinal symptoms scale assessment**

The CARS was used to diagnose and evaluate the severity of clinical symptoms of ASD patients. The CARS consisted of 15 items rated on a 7-point scale from one to four; higher scores are associated with a higher level of impairment. Total scores can range from a low of 15 to a high of 60; scores below 30 indicate that the individual is in the non-autistic range, scores between 30 and 36.5 indicate mild to moderate autism, and scores from 37 to 60 indicate severe autism. We further categorized these items into three functional and emotional CARS domains: *Social impairment*, *Negative emotionality*, and *Distorted sensory response* ^16^. ADOS was used as a supplement to gauge disease severity, and it contained total score items and 4 sub-items for assessment of *Social interaction*, *Communication*, *Play*, and *Imaginative use of materials* for individuals suspected of having ASD ^17^.

The questionnaire assessments about feeding behaviors and gastrointestinal symptoms were carried out on ASD patients. For feeding behaviors, we focused on two aspects: “narrowed food spectrum” and “resistance to accept new food”. Furthermore, we assessed gastrointestinal symptoms on five dimensions: abdominal pain, vomiting, abdominal distension, constipation and diarrhea, in which any item has positive score the patient is considered as having gastrointestinal problem.

**Faeces sample collection and data analysis**

***DNA extraction, 16S rRNA gene amplicon, and sequencing***

The feces samples were stored at −80 °C until DNA extraction. The DNA was extracted from 200 mg samples using the E.Z.N.A Stool DNA Kit (Omega BioTek, Inc., GA, USA) following the manufacturer’s instructions. Polymerase chain reaction (PCR) amplification of 16S rRNA genes was performed using general bacterial 341F (5’-CCTACGGGNGGCWGCAG-3’) and 805R (5’-GACTACHVGGGTATCTAATCC-3’). The PCR products were indexed and mixed at equal ratios for 2x300 bp paired-end sequencing using a MiSeq v3 Reagent Kit. The MiSeq platform (Illumina) was used, and the sequencing was performed at Tiny Gene Bio-Tech (Shanghai) Co., Ltd.

***16S rRNA sequencing analysis***

After quality filtering and primer sequence removal, all sequences were analyzed using the Quantitative Insights into Microbial Ecology software package (QIIME 2, versions 2018.8) following the suggested workflow ^18^. The fragments were inserted into the 16S rRNA gene context, alignments were generated by the MAFFT program ^19^, and a phylogenetic tree was obtained from the masked alignments using FastTree ^20^. Taxonomic analysis was performed using the Naive Bayes classifier (q2-feature-classifier plugin) trained on GreenGenes 13_8 99% operational taxonomic units (OTUs) full-length sequences. Alpha diversity was analyzed using the core-metrics-phylogenetic for observed diversity metrics including the evenness diversity metric, Faith’s phylogenetic diversity metric, chao1 richness estimator, and Shannon’s entropy. Estimates of within-sample diversity were made at a rarefaction depth of 10,000 reads per sample. Between-group differences in alpha diversity were evaluated using the Kruskal-Wallis test. Principal coordinate analysis (PCoA) was conducted based on the Bray-Curtis dissimilarity.

***Differential abundance analysis***

Group differences were investigated using Kruskal-Wallis test to determine the relative abundance of bacteria at the genus level. The microbial gene content of samples was inferred from 16S rRNA gene sequencing data using PICRUSt2 ^21^. PICRUSt2 expands PICRUSt and includes a larger reference genome database and also provides MetaCyc ^22^ pathway predictions. Group differences in predicted pathways were identified using edgeR in *R*. *P* values were adjusted for multiple hypothesis testing using the Benjamini–Hochberg false discovery rate (FDR) procedure.

**Imaging data pre-processing and analysis**

***Image acquisition***

Participants were scanned using a Siemens Verio 3.0-Tesla MRI scanner (Siemens Medical Solutions, Munich, Germany) or Philips Inginia 3.0-Tesla MRI scanner, with a 32-channel head coil and four-channel neck coil. Earplugs, earphones, and extra foam padding were provided to the participants to reduce the impact of the sound of the scanner during the scan. A high-resolution anatomical T1-weighted magnetization‐prepared rapid gradient echo image (Siemens: 192 sagittal slices; voxels = 1 × 1 × 1 mm; repetition time [TR] = 2300 ms; echo time [TE] = 2.28 ms; inversion time = 1100 ms; flip angle = 8°, field of view = 192 × 192 × 192 mm; Philips: 170 sagittal slices; voxels = 1 × 1 × 1 mm; TR = 7900 ms; TE = 3.5 ms; inversion time = 1100 ms; flip angle = 7°, field of view = 250 × 193 × 170 mm) was acquired. The scanner effect was controlled as the covariate during analysis.

***Image pre-processing***

T1-weighted images were processed using statistical parametric mapping SPM12 software (<http://www.fil.ion.ucl.ac.uk/spm/>) with the computational anatomy toolbox CAT12, and incorporating the Diffeomorphic Anatomical Registration Through Exponentiated Lie Algebra (DARTEL) toolbox. The steps included segmentation, registration, normalization, and smoothing. Notably, the Template-O-Matic Toolbox was used to customize tissue probability maps (TPMs) for a 4.75-year-old that matched the age of the participants with images in this study. These customized TPMs were used for the initial spatial registration and segmentation. A standard optimized method of iterative tissue segmentation and spatial normalization, using both linear (12-parameter affine) and non-linear transformations, was performed. So that the residuals in later analyses conformed more closely to a Gaussian distribution and to account for individual differences in brain anatomy, the modulated grey matter images in the MNI space were smoothed with an isotropic Gaussian kernel of 8 mm full-width at half maximum. The resulting voxel size was 1.5×1.5×1.5 mm^3^. Total gray matter volumes of cerebral regions were extracted based on the automated anatomical labeling (AAL) atlas, and the sum of the volumes was used for subsequent analyses.

**Statistical analysis**

***Definition of signature associated with severity of ASD***

We proposed a new computational pipeline to categorize ASD subjects using the gut microbiota signature. The hypothesis is that patients with similar behavior characteristics tend to have similar gut microbial patterns. This can be formulated as a similarity maximization problem:

$\underset{(g_{1},g_{2}\cdots,g_{m})}{arg \max} \frac{cov(f\left( X \right),f(Y))}{\sigma_{f(X)}\sigma_{f(Y)}}$ (1)

where *cov,* $\sigma$ are the covariance and standard deviation of the rank variables, respectively, and *f* is Bray-Curtis metric function. X presents the behavioral scale matrix, and Y is gut microbial matrix, in which features ($s_{i}$ denotes the *i*-th item of behavioral scale, $g_{j}$ denotes the *j*-th taxa of microbiota) in rows and individual samples in columns. Taxa were excluded if they were not present in at least 5% of samples on a genus level. After Wisconsin double standardization, the Bray-Curtis metric function was used to generate two distance matrixes: the microbiota-related similarity matrix and the behavior-related similarity matrix (including CARS total score and 15 subscales). To obtain a maximal correlation with the behavior-related similarity matrix, the microbiota-related similarity matrix under different taxa combinations was changed to search for an optimal solution. A greedy strategy was adopted to screen the taxa combination, in which each iteration removed taxa to increase the Spearman correlation of two matrices until the correlation revealed no more changes. Hierarchical clustering using the average linkage of the identified microbial combination was employed to visualize sample clusters.

***Analysis of clinical symptoms***

When ASD subpopulations were determined, the inter-group differences in behavioral measurements (i.e., CARS total score, ADOS total score) were then evaluated by the Kruskal-Wallis test. A permutation test was conducted by performing 5000 random permutations of the subpopulation label. We further examined the differences in three behavioral and emotional domains of the CARS between ASD subpopulations using the Kruskal-Wallis rank-sum test, and the domain correction was considered by permutation test (5000 random permutations). The effect size of the Kruskal-Wallis test was evaluated by Epsilon square ($\varepsilon^{2}$). The proportion of severe subjects was defined as the severe-ratio, and the severe-ratio difference between subpopulations was evaluated by Fisher’s exact test. The differential analysis for feeding behaviors and gastrointestinal symptoms were carried out by the Kruskal-Wallis rank-sum test and Fisher’s exact test.

***Analysis of brain volumes***

To investigate the possible influence of the microbial signature on the brain, we integrated the brain structural information and explored the difference in brain volumes between the ASD subpopulations. Permutation-based Student’s *t*-test (https://github.com/lrkrol/permutationTest) was established by 5000 random permutations to control the family-wise error rate, after removing the confounding effects of age, gender, and scanners. Then, permutation test (5000 random permutations) based on Spearman’s rank coefficient was applied to investigate the associations between behavioral symptoms and the brain volumes identified above in these two subpopulations.

***Validation in independent test sets***

To validate the recurrent pattern, we trained a model by performing *k*-nearest neighbor (*k*NN) analysis on the microbial signature with leave-one-out cross-validation. The microbial pattern and difference of behavioral assessments were validated by the training model on the independent test set and two public gut microbiota data sets (SRP093968, PRJEB15418), in which the SRP093968 set included 30 children with ASD from India, and the PRJEB15418 set consisted of 40 patients with ASD from Italy.

**Supplementary table S1**


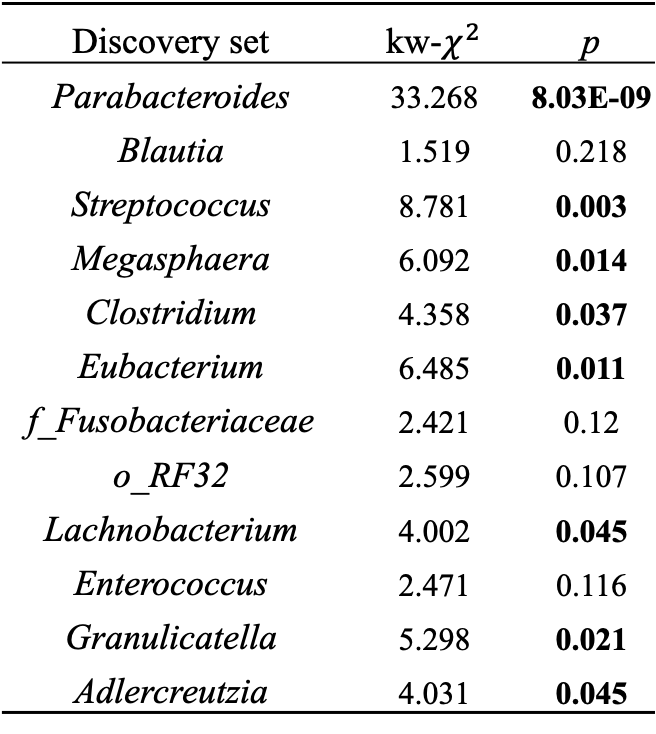

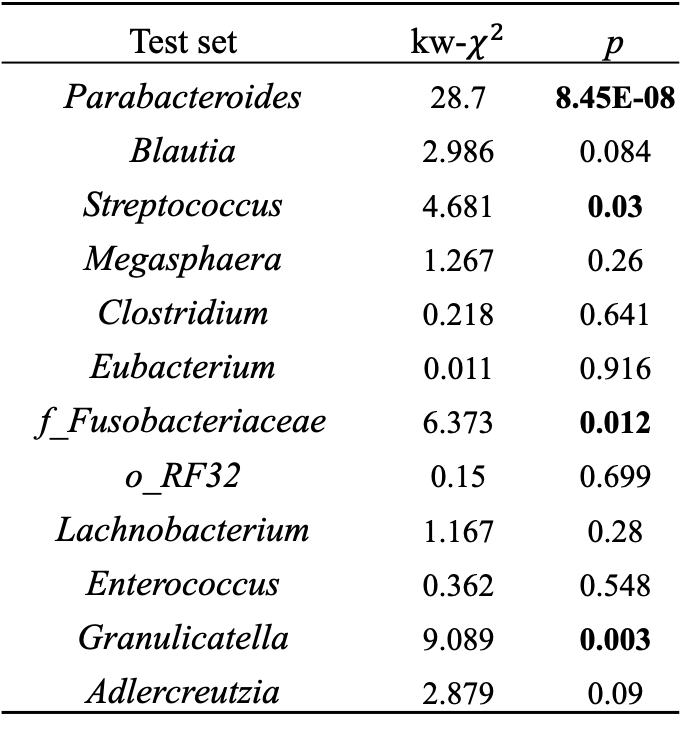


Supplementary table S1: The difference of 12 genera between two subpopulations in the discovery and test sets.

**Supplementary table S2:**


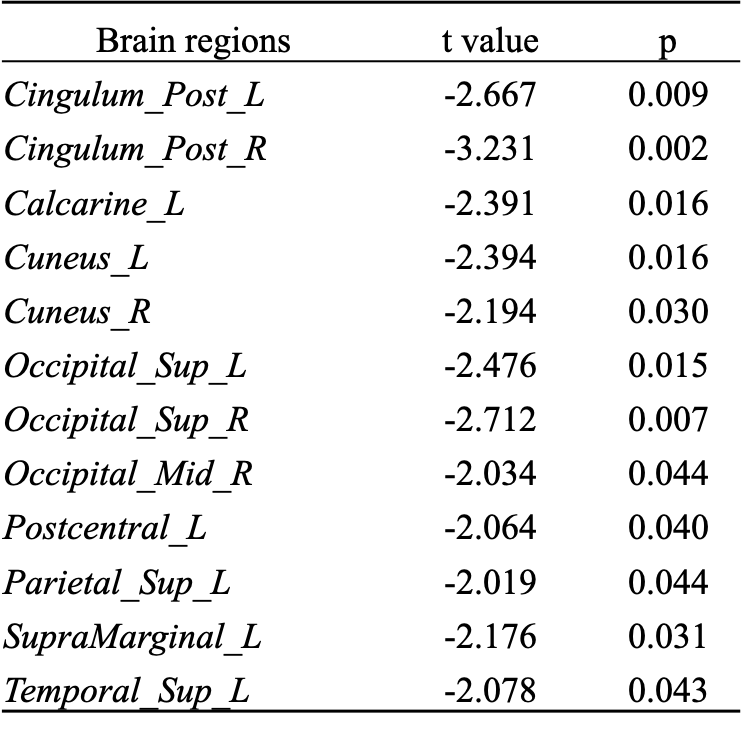


Supplementary table S2: Brain regions with grey matter volumes differed between ASDmp1 and ASDmp2 groups, after permutation-based family-wise-error correction (FEW < 0.05). *p* value is based on 5000 random permutations.

**Supplementary table S3**


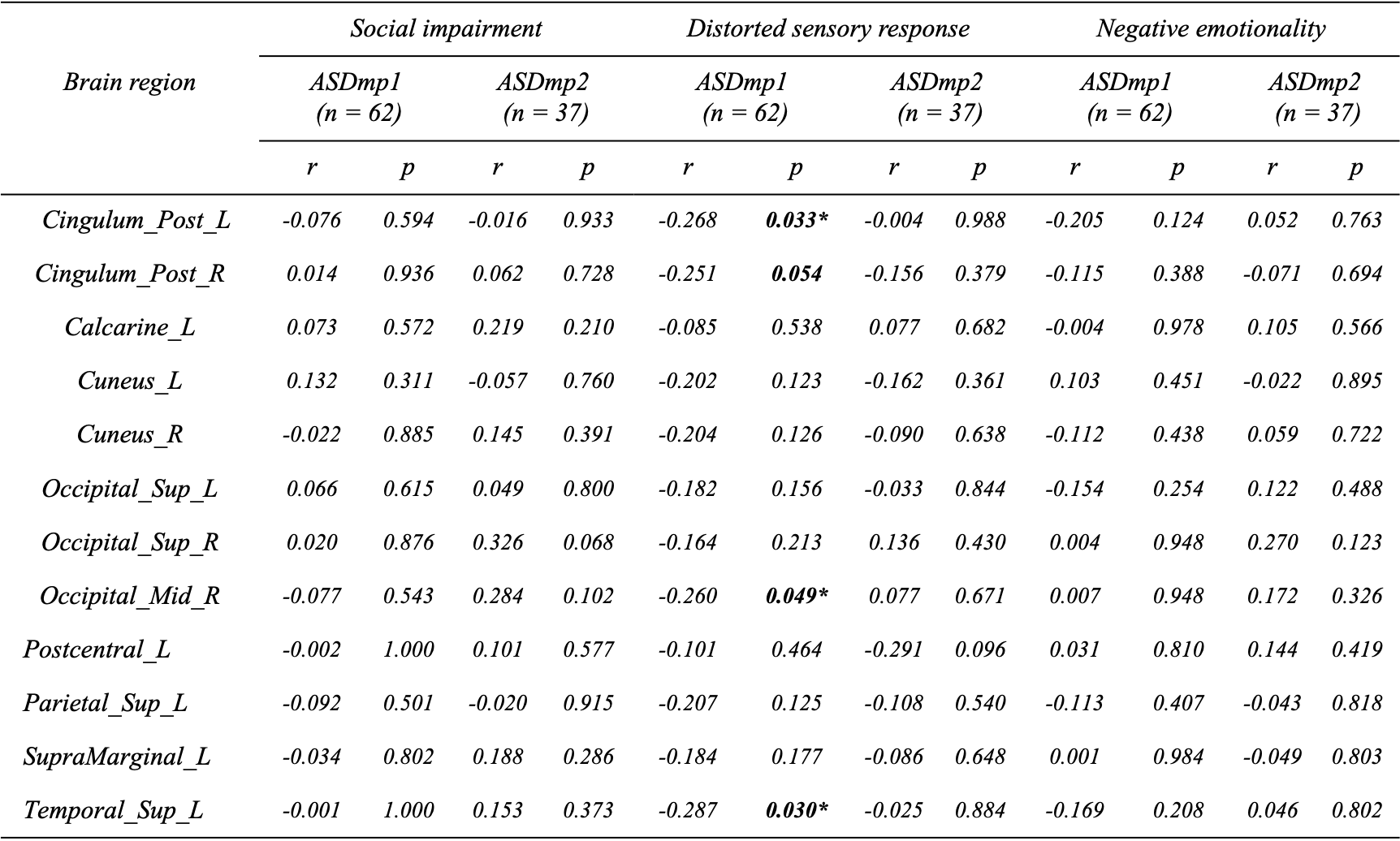


Supplementary table S3: Associations of brain structure differences with behavior severity in ASD^mp1^ and ASD^mp2^ subpopulations. *p* value is based on 5000 random permutations.

**Supplementary** **Figure S1**

**Figure S1** Discovery set AUC reflects the 12-taxa signature for discriminating ASD^mp1^ and ASD^mp2^

**Supplementary** **Figure S2**

**Figure S2** $\alpha$-diversity

**Supplementary** **Figure S3**

**Figure S3** The average relative abundance of 12 taxa signature in public data set
